# Clinical Recommendations for Remote Robotic Assisted Surgery From the CRSA 2025 International Consensus Conference

**DOI:** 10.1002/wjs.70468

**Published:** 2026-07-14

**Authors:** Yuman Fong, Dimitri A. Raptis, Louis Kavoussi, Sherry M. Wren, Dennis Fowler, Mehran Anvari, Francesco Bianco, Xiao Liang, Zheyong Li, Xiaoyu Yin, Micaela Piccoli, Saad Aldousari, John Marks, Baiyong Shen, Clifford Ko, Jelle Ruurda, Barbara Seeliger, Jeroen Hagendoorn, Rahila Essani, R. Matthew Walsh, Dmitry Oleynikov, Stephanie B. Jones, Vivek Bindal, Hyun Koo Kim, Woo Jin Hyung, Laleh Melstrom, Daniel Hashimoto, Francesco Celotto, Liaohai Leo Chen, Bryan M. Burt, Kelly Lafaro, Fernando Elli, Yusef Kudsi, Francesca Ratti, Erik Wilson, Paolo Pietro Bianchi, Annette Morgan, Stephen T. Bartlett, Laila Rashidi, Liu Yu, Bill Gawkins, Piet Hinoul, Bill Peine, Yanghee Woo, Mustafa Raoof, Daniel Jones, Supriya Deshpande, Jordana Bernard, Yulun Wang, Dieter C. Broering, Pier Cristoforo Giulianotti

**Affiliations:** ^1^ City of Hope National Medical Cancer Center Duarte California USA; ^2^ King Faisal Specialist Hospital and Research Centre Alfaisal University Riyadh Saudi Arabia; ^3^ Northwell New Hyde Park New York USA; ^4^ Stanford University Palo Alto California USA; ^5^ Sovato Health Santa Barbara California USA; ^6^ McMaster University Hamilton Ontario Canada; ^7^ University of Illinois Chicago Chicago Illinois USA; ^8^ Sir Run Run Shaw Hospital Zhejiang University Hangzhou China; ^9^ Alaer Hospital XPCC Xinjiang China; ^10^ The First Affiliated Hospital of Sun Yat‐sen University Guangzhou China; ^11^ Ospedale Civile Baggiovara Azienda Ospedaliero Universitaria di Modena Modena Italy; ^12^ Kuwait University Sabah Al‐Ahmad Urology Center Kuwait Kuwait; ^13^ Lankenau Medical Center Main Line Health Wynnewood Pennsylvania USA; ^14^ Ruijin Hospital Shanghai Jiaotong University Medical School Shanghai China; ^15^ University of California Los Angeles California USA; ^16^ Utrecht University Medical Center Utrecht the Netherlands; ^17^ Department of Digestive and Endocrine Surgery University Hospitals of Strasbourg Strasbourg France; ^18^ IHU Strasbourg, Institute of Image‐Guided Surgery Strasbourg France; ^19^ ICube, UMR 7357, CNRS, INSERM U1328 RODIN, University of Strasbourg Strasbourg France; ^20^ Advent Health Celebration Florida USA; ^21^ Cleveland Clinic Cleveland Ohio USA; ^22^ Monmouth Medical Center Long Branch New Jersey USA; ^23^ Max Healthcare New Delhi India; ^24^ Korea University Seoul South Korea; ^25^ Yonsei University College of Medicine Severance Hospital Seoul South Korea; ^26^ University of Pennsylvania Philadelphia Pennsylvania USA; ^27^ Department of Surgical Oncological and Gastroenterological Sciences University of Padova Padova Italy; ^28^ Worcester Polytechnic Institute Worcester Massachusetts USA; ^29^ Johns Hopkins University Baltimore Maryland USA; ^30^ Mayo Clinic Jacksonville Florida USA; ^31^ Brigham and Women's Hospital Boston Massachusetts USA; ^32^ IRCCS San Raffaele Scientific Institute Milano Italy; ^33^ Vita‐Salute San Raffaele University Milano Italy; ^34^ Memorial Hermann‐Texas Medical Center Houston Texas USA; ^35^ Università di Milano Milan Italy; ^36^ MultiCare Health System Tacoma Washington USA; ^37^ Microport Medbot Shanghai China; ^38^ Johnson and Johnson New Brunswick New Jersey USA; ^39^ Virtual Incision Corporation Lincoln Nebraska USA; ^40^ Medtronic Ashland Massachusetts USA; ^41^ Rutgers New Jersey Medical School Newark New Jersey USA

**Keywords:** danish model of consensus, medical ethics, regulatory approval, remote robotic surgery, remote surgery, review, robotic workflow, robotic‐assisted surgery, telesurgery

## Abstract

**Background:**

Implementation of remote robotic‐assisted surgery (rRAS) has the potential to significantly improve access to high‐quality surgical care for patients worldwide. Advances in robotic systems and telecommunications now enable highly reliable, low latency, and cybersecure connectivity. These technological developments, together with the broader adoption of telemedicine, have driven an increasing interest in the implementation of rRAS.

**Methods:**

The Clinical Robotic Surgery Association (CRSA) convened a conference to develop international clinical recommendations for rRAS using the Danish Model of Consensus. The society identified five key issue categories and invited a panel of experts to address specific questions for each. Panel members and a jury of 10 unbiased, non‐expert in rRAS professionals participated in a day‐long conference on November 19, 2025 (Los Angeles, USA). During the conference, participants presented, discussed, and reached consensus on specific guidelines relevant to each category. Immediately after the conference, the jury reviewed and approved the recommended guidelines.

**Results:**

Multiple recommendations were approved for each of the following categories: Operating Models, Roles and Responsibilities (2 recommendations), Clinical Pathway (2 recommendations), Emergency Management (8 recommendations), Technical Considerations (5 recommendations), and Medico‐legal and Ethical Considerations (4 recommendations).

**Conclusions:**

There was consensus that with recent advances in telecommunication and robotic technologies, rRAS can be offered safely and effectively if the identified safeguards are realized. Adoption of rRAS is expected to continue advancing rapidly.

## Graphical abstract

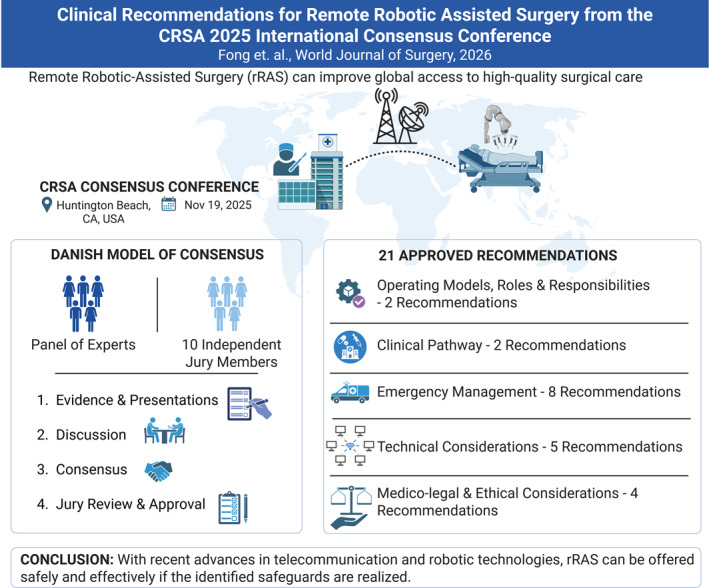

## 1 Introduction

Robotic‐assisted surgery (RAS) was conceived with remote workflow as a central objective. Contemporary RAS separates the surgeon from the patient by an interceding computer interface and effector controllers. Extending this model to true remote systems introduces unique requirements to safely mitigate physical distance between the surgeon and the patient. This requires a low latency, cybersecure communications network capable of complex surgical situational awareness. Early efforts demonstrated the technical feasibility of remote‐RAS (rRAS) [1, 2]; however, widespread adoption was limited by communication costs and technical, legal, and system design challenges.

During the last 2 decades, RAS has become an integral part of routine surgical practice. Currently, it is widely used across multiple surgical disciplines, including urology, gynecology, general, upper gastrointestinal, hepatobiliary, transplantation, orthopedics, thoracic, pediatric surgery, hernia, and others [3]. The adoption of RAS is expected to expand because of superior patient outcomes [4, 5, 6], shorter learning curves [7], lower conversion rates [4], and superior ergonomics [8] compared with conventional laparoscopic or thoracoscopic approaches, benefiting patients and surgeons.

Three developments have given prominence to rRAS: First, telecommunications infrastructure is cheaper with improved reliability and cybersecurity [9]. Second, robotic systems have reduced inherent latency and are designed for remote workflows [10, 11]. Third, escalation of telemedicine during the COVID‐19 pandemic, coupled with adoption of automation and remote operations in various industries, has increased public acceptance of remote surgery [12].

Over 500 rRAS cases have been performed in humans worldwide, occasionally with surgeons located thousands of miles from patients [11, 13]. These procedures have established safety of rRAS in expert hands, which has prompted regulatory approval of a tele‐robotic surgical system in China [14]. Recently, a randomized trial of remote versus local robotic surgery demonstrated non‐inferiority of rRAS for prostatectomy and partial nephrectomy [15]. rRAS is thus gaining momentum.

Several groups have published recommendations for rRAS, including the Japan Surgical Society [16], the Society of Robotic Surgery [17], and technical guidelines from a joint industry consortium [18]. The objective of this study was to develop internationally accepted rRAS recommendations based on consensus by a multidisciplinary panel of experts in the field and an independent jury, using the rigorous Danish Consensus Model.

## 2 Methods

This project was conducted by the Clinical Robotic Surgery Association (CRSA) to develop clinical consensus‐based international recommendations for rRAS, using the Danish Model of Consensus (Supporting Information S1: Appendix Figure A1). The Remote Robotic‐Assisted Surgery International Consensus Conference (RICC) was held on November 19, 2025, (Huntington Beach, California) to coincide with the World Congress of the CRSA.

### 2.1 Preparatory Work

Prior to the RICC, experts in surgery, robotics, communications, hospital administration, and health policy identified key factors that could influence the safety and outcomes of rRAS. Identified issues were grouped into five categories: (1) operating models, roles, and responsibilities, (2) clinical pathway, (3) emergency management, (4) technical considerations, and (5) medico‐legal and ethical considerations. The entire process was coordinated by CRSA administration and the editorial team at the Department of Surgery, City of Hope Cancer Center. CRSA leadership invited experts with relevant clinical, scientific, and technical expertise from 14 nations to serve on the panels.

Each panel was assigned a lead member and consisted of 8–12 surgeons and specialists with direct experience in developing, implementing, or studying remote robotic surgery programs. All panel members met over several video conferences to review the literature, define questions, and draft consensus statements, resulting in 16 scientific questions and 21 statements.

Literature research was conducted by the editorial team between March and June 2025 using Ovid MEDLINE, Embase, Scopus, Google Scholar, ClinicalTrials.gov, and the Cochrane Central Register of Controlled Trials, using team‐identified keywords. Only English language articles were included. Reviews were performed according to PRISMA guidelines [19], and each panel screened and included relevant studies followed by further cross‐referencing. Evidence was based on a 2025 systematic review summarized in a previous editorial [20], and, for technical considerations on a previous consensus‐based guidelines by key MedTech leaders [18]. Relevant literature, which included 229 articles, is listed in Supporting Information S1: Appendix Table A2.

The Grading Recommendations, Assessment, Development and Evaluations (GRADE) approach was used for a transparent framework and comprehensive analysis and rating of evidence [21]. Effect sizes of results and potential limitations (bias risk, imprecision, indirectness, inconsistency, and publication bias) were considered [22]. Considering the limited number of large series or randomized trials, a modified GRADE approach was applied for reporting effect size narratively [23]. Expert recommendations were graded (strong or conditional, and “for or against” each question, based on five decision domains of the GRADE approach: (1) Estimated effects (balance between desirable and undesirable outcomes), (2) Quality of evidence for outcomes (confidence in the effect on important outcomes), (3) Confidence in values and preferences, and their variability (similar across the target population), (4) Resource implications (are resources worth the benefit), and (5) Overall Quality of Evidence [24].

### 2.2 Consensus Process

The Danish model of consensus was selected for its participatory approach and ability to reduce expert bias through input from non‐experts or other participants who may bring personal or ethical perspectives [25, 26, 27]. During the conference, each of the 5 panels presented 30‐min structured evidence and graded recommendations, followed by 60‐min audience discussions. Recommendations were then subjected to formal voting, requiring a predefined threshold of ≥ 80% agreement for acceptance. Statements that did not meet this threshold were modified in real time during the consensus process until agreement was reached or were excluded if consensus could not be achieved. To avoid potentially leading statements (i.e., prompting or encouraging the desired vote), GRADE approach‐based recommendations were anonymously and mandatorily voted with either yes or no response.

An independent jury (as required by the Danish model) of 10 high‐profile individuals (Table 1) from other areas of scientific, clinical, or methodological backgrounds, and where appropriate, representative of broader stakeholders and patient perspectives, but without expertise in remote surgery, observed the presentations, discussions, and voting. Following the conference, the jury met independently with the RICC writing committee to discuss, modify, or adopt each recommendation, informed by discussions and voting results, mitigating bias and giving a platform to the entire audience in shaping the recommendations. The jury unanimously agreed with the final recommendations and consented to publication. The entire process led to the development of 21 international recommendations based on group consensus (Table 2).

**TABLE 1:** List of jury members.

Jury			
Cristina Ferrone^a^	Surgeon/Administrator	USA	Cedars Sinai medical center
Sidra Haye	Epidemiology	USA	University of California Los Angeles
Jaime Reiter	Nursing administration	USA	University of Southern California
Kimberly small	Patient advocate	USA	Los Angeles
Paolo Fiorini	Basic robotic engineer	Italy	University of Verona
Taryne Imai	University surgeon	USA	University of Hawaii
David Bentrem	University surgeon	USA	Northwestern University
Victoria O'Connor	Managed care surgeon	USA	Kaiser permanente
James Peake	Surgeon	USA	Former secretary Veterans administration and surgeon general US army
Roland de Wilde	Data scientist/Surgeon	Netherlands	Erasmus medical center

^a^ Head of Jury.

**TABLE 2:** Expert panel guidelines, quality of related evidence and GRADE of recommendation, and voting results.

	Panel 1: Operating models, roles, and responsibilities	Expert panel vote	Jury vote
1	Members of the remote robotic surgery care team include remote surgeon, patient site credentialed practitioner, circulating nurse, scrub nurse/technician, and anesthesiologist/nurse anesthetist. The entire team shall undergo training and qualification for remote robotic surgery procedures. (Quality of evidence: Moderate | grade of recommendation: Strong)	100%	100%
2	Medical liability and responsibility for adverse outcomes during or after remote robotic surgery should be shared by all members of the team. For remote surgeons, liability may be tied to their role in directing the procedure, regardless of whether they are remotely controlling the instruments. (Quality of evidence: Low | grade of recommendation: Strong)	76%	100%
	**Panel 2: Clinical pathways**		
3	The preoperative consultation process must establish protocols allowing the remote surgeon access to the patient's health records and imaging. Institutions must implement a standardized pre‐operative pathway for remote robotic surgery that includes a remote–local joint consultation, validated network, and equipment inventory as well as testing. (Quality of evidence: Low | grade of recommendation: Strong)	100%	100%
4	Responsibilities should be decided, agreed upon by the entire team, and communicated to the patient prior to the day of remote robotic surgery. (Quality of evidence: Very low | grade of recommendation: Strong)	100%	100%
	**Panel 3: Emergency management**		
5	Remote procedures should be classified at the time of scheduling based on their risk of requiring an emergency conversion to open surgery for hemorrhage control or a critical change in patient clinical status requiring expedited completion of the procedure. (Quality of evidence: High | grade of recommendation: Strong)	92%	100%
6	Remote robotic surgical procedures should have a credentialed local surgeon capable of performing the procedure or hemorrhage control. For procedures classified as high risk, the credentialed surgeon should be in the patient operating room during the critical portions of the procedure. For moderate‐ and low‐risk procedures, the credentialed surgeon should be physically onsite and available throughout the procedure. (Quality of evidence: Low| grade of recommendation: Strong)	92%	100%
7	The remote robotic surgery patient site must be equipped with surgical instruments appropriate to perform the scheduled procedure as an open/laparoscopic operation or an emergency open surgery damage control procedure. Minimum requirements should include instrumentation appropriate for the body cavity, vascular clamps and sutures, and adjunctive hemostatic products. For high‐risk procedures, case‐specific instrumentation and supplies for open surgery must be immediately available. For moderate‐ to low‐risk procedures, minimum requirements include instrumentation appropriate for the body cavity must be available on site in the operating suite complex. (Quality of evidence: Low| grade of recommendation: Strong)	92%	100%
8	Patients scheduled for procedures classified as high‐risk of hemorrhage should undergo blood type and cross‐matching and have confirmed products available at the patient site. Patient site facilities must have O‐negative and/or type‐specific blood products available on site. For ambulatory surgery patient site centers or moderate‐to‐low‐risk procedures, availability of blood transfusion products should be according to institutional regulation. (Quality of evidence: High | grade of recommendation: Strong)	100%	100%
9	If remote robotic surgery is performed in a patient site setting that is an ambulatory or non‐tertiary/referral hospital, a formalized transfer protocol must be in place to ensure rapid, safe transport to a definitive inpatient care facility. (Quality of evidence: High | grade of recommendation: Strong)	100%	100%
10	A joint emergency management plan must be in place at the patient‐site and remote robotic surgical site. The plans must include communication and management protocols for critical changes in the medical status, uncontrolled hemorrhage, and technology failures (robot and/or communication). (Quality of evidence: Moderate | grade of recommendation: Strong)	97%	100%
11	A remote robotic surgery‐specific preoperative briefing should be performed in addition to the standard protocol briefing. (Quality of evidence: High | grade of recommendation: Strong)	94%	100%
12	All patient site care teams including primary and remote robotic surgeons shall participate in simulation training, including emergency undocking, emergency conversion for bleeding, technology/communication failure, and crisis resource management, before initiating a remote robotic surgery program and shall participate in periodic standardized training. (Quality of evidence: High | grade of recommendation: Strong)	94%	100%
	**Panel 4: Technical considerations**		
13	The surgical‐grade network must deliver predictable, consistent, and deterministic performance for the intended remote robotic surgical procedure: (1) Highly reliable using proactive monitoring and reporting, network redundancy, and automated failover to provide 99.999% uptime; (2) Guaranteed bandwidth for the duration of the planned procedure; (3) Optimal total teleoperation latency < 300 ms, ideally under 200 ms to meet the clinical requirements of the intended procedure; (4) Minimal or no jitter and zero or near‐zero packet loss (quality of evidence: Moderate | grade of recommendation: Strong)	92%	100%
14	The surgical‐grade network and the remote robotic surgical system must use the IP protocol for data transmission and to authenticate and authorize connected devices, personnel, and locations for each specific remote robotic surgical procedure. (Quality of evidence: Low | grade of recommendation: Strong)	97%	100%
15	All data transmitted from device to device across the surgical‐grade network, including video, audio, and control signals, must be secure by design, use end‐to‐end encryption, and comply with cyber‐physical security and privacy requirements set by relevant regulatory authorities and institutional policies. Any data breach/cybersecurity incident should be reported immediately between both remote robotic surgical sites. (Quality of evidence: High | grade of recommendation: Strong)	100%	100%
16	Telepresence (audiovisual) communications must meet the same network performance standards as remote robotic surgery video and control systems. The remote physician should have available an effective, high‐resolution view of the patient, key participants, and critical monitors, with two‐way audio at the patient site, while patient‐site participants should have an effective view of the remote physician. (Quality of evidence: Low| grade of recommendation: Strong)	100%	100%
17	The remote robotic surgery system must maintain clinical performance and safety mitigations despite latency and other challenges of remote care and must notify the on‐site and remote teams of changes in the network, energy controls, or other conditions that could affect performance. (Quality of evidence: Low| grade of recommendation: Strong)	100%	100%
	**Panel 5: Medico‐legal and ethical considerations**		
18	The patient should be consented for the risks and benefits of the specific surgical procedure. Informed consent for remote robotic surgery should explicitly describe the configuration of the remote surgical setup (model of care), identify all responsible parties and their respective roles; disclose network and equipment risks and the impact on patient safety; alternative approaches, and outline emergency contingency procedures including conversion. The rationale for selecting a remote approach should be transparent. (Quality of evidence: Low | grade of recommendation: Strong)	94%	100%
19	Institutions planning on initiating a remote robotic surgical program should use the recommendations in this document (and the consensus technical guideline—reference guideline here) to determine their readiness and process standards before performing remote robotic surgery. Remote robotic surgery should be practiced through regulated, monitored, and ethically transparent processes. (Quality of evidence: Low | grade of recommendation: Strong)	78%	100%
20	Institutions shall privilege surgeons and other clinicians specifically for remote robotic surgery. (Quality of evidence: Moderate | grade of recommendation: Strong)	100%	100%
21	Until regulatory clearance, a given remote robotic surgery program should obtain Institutional review board (IRB), ethical committee, or equivalent approval. Each case should be prospectively recorded in a registry. Registries could capture technical, clinical, and network performance data for evaluation, research, safety surveillance, and benchmarking. (Quality of evidence: High | grade of recommendation: Strong)	97%	100%

## 3 Results

Jury‐approved clinical guidelines are presented in Table 2. A brief discussion from each panel is presented below.

### 3.1 Panel 1: Operating Models, Roles, and Responsibilities

Panel 1 reviewed the various operating models for rRAS (Table 3), in addition to previous definitions (Supporting Information S1: Appendix Table A1), pre‐clinical, and human data. Technical safety of rRAS has been established in large animal models [1, 28].

**TABLE 3:** Operating models for remote robotic‐assisted surgery.

	Teleproctoring (observation only)	Telementoring/Teleprecepting without instrument control	Telementoring/Teleprecepting with instrument control	Remote robotic‐assisted Co‐surgery	Remote robotic‐assisted Co‐surgery with planned conversion	Full remote robotic‐assisted surgery
Control of robots	Patient site credentialed practitioner	Patient site credentialed practitioner	Shared operating authority	Shared operating authority	Shared operating authority	Remote credentialed practitioner
Patient Responsibility	Patient site credentialed practitioner	Patient site credentialed practitioner	Patient site credentialed practitioner	Patient site credentialed practitioner	Patient site surgeon	Remote surgeon
Emergency response			Patient site credentialed practitioner	Patient site credentialed practitioner	Patient site credentialed practitioner	Emergency response practitioner

Many studies have demonstrated safe and successful rRAS in humans, for for example, a transatlantic cholecystectomy using the Zeus Robot (Computer Motion, Santa Barbara, CA) [1, 29, 30, 31]. Availability of cheaper and reliable communication systems, evolution of telemedicine, [32], evolution of surgical robots have increased human studies in the last decade (Table 4) [9, 10, 11, 13, 33, 34, 35, 36, 37, 38, 39, 40, 41, 42, 43]. Several urologic, gastrointestinal, hepatobiliary procedures have been performed safely with low morbidity and no reported mortality across national and continental boundaries [11, 41, 42, 44]. The National Medical Products Administration (NMPA) of China has now approved a surgical workflow for rRAS [14].

**TABLE 4:** Current human remote robotic‐assisted surgery studies and reported complications.

Study/[PMID]	Procedure	Robotic system	N	C	Complications
Bauer et al. 2001/[11886670] Baltimore‐Rome 4500 miles	Remote percutaneous renal access	1) PAKY needle driver 2) RCM robot	1	0	None (accurate access)
Marescaux et al. 2002/[11923603] New York‐Strasbourg > 14,000 km	Remote robot‐assisted laparoscopic cholecystectomy	The ZEUS system (computer motion)	1	0	None
Bove et al. 2003/[12803985] Baltimore‐Rome 9230 km	Basic: Spermatic vein ligation 8 Retroperitoneal renal biopsy 2 Percutaneous approach to kidney 3 Advanced: Nephrectomy 3 Pyeloplasty	1) AESOP, for orientation of the laparoscope 2) PAKY, for percutaneous renal access.	17 5: Not possible	2	Pyeloplasty: Severe dysrhythmia Nephrectomy: Several adhesions made adequate isolation of kidney impossible
Anvari et al. 2005/[15729068] Hamilton‐North Bay 400 km	Laparoscopic surgeries	Zeus TS microjoint system	21	0	No serious complications
Patel et al. 2019/[31709402] Ahmedabad, India‐Gandhinagar 20 miles	Robotic‐assisted percutaneous coronary intervention (R‐PCI)	The CorPath GRX robotic system (Corindus Vascular robotics)	5	0	None
Rogers et al. 2020/[39460826] Beijing‐Hainan 2600 km	A triple‐console, robot‐assisted radical prostatectomy	Kangduo endosopic surgical Robot (KD‐SR‐01, sagebot medical)	1	0	No intra‐operative or postoperative complications
Tian et al. 2020 [32252160] Beijing Jishuitan Hospital‐6 different hospitals in China	5G telerobotic spinal surgery	TiRobot system	12	0	No intra‐operative adverse event was found
Zhou et al. 2025/[39557365] Lanzhou, China 52 km	5G remote urological surgeries	Toumai surgical system	14	0	No intra‐operative or postoperative complications
Li et al. 2023/[35817641] Qingdao‐8 hospitals. Median distance 187 km	Robot‐assisted laparoscopic radical nephrectomy	Micro hand S robotic system (shangdong WEGO surgical Robot Company)	29	0	No major postoperative morbidities. 1 transfusion. 2 wound infection, 1 intestinal obstruction
Yang et al. 2022 [35476791] Qingdao ‐anshun ∼3000 km	Ultra‐remote radical cystectomy	MicroHand endoscopic surgical robot system	1	0	No intra‐operative complications
Yang et al. 2024/[38238690] Nanjing medical University‐Xinjiang Kezhou > 5800 km	Percutaneous nephrolithotomy (PCNL)	RTDS (MGIUS‐R3, Huada Cloud‐shadow medical technology co.)	15	—	No complications of grade III‐V were found 2 patients with grade II bleed: 1 required readmission and transfusion
Tian et al. 2024/[39831214] Shanghai‐Kashi > 5000 km	Right upper lobectomy and lymph node dissection	Toumai endoscopic surgical robot system (MicroPort, Shanghai, China)	1	0	None reported
Wang et al. 2024/[37977963] Beijing‐Sanya > 6000 km	Renal cancer, prostate cancer, adrenal tumor.	Edge medical Robot (MP1000)	6	0	No Clavien‐Dindo > grade II observed at 2‐week follow‐up
Moschovas et al. 2024/[39226445] Beijing‐Harbin 1300 km	Telesurgery robotic‐assisted radical prostatectomy	Edge medical Robot (MP1000)	1	0	No intra‐operative or postoperative complications
Fan et al. 2025/[39744935] Hangzhou, Zhejiang—Alaer city, Xinjiang 4670.2 km	Ultra‐remote hepatobiliary and pancreatic surgery	Toumai four‐arm robot (Model:MT‐1000)	5	0	1 instrument adverse event 1 postoperative complication (Grade I)
Aldousari et al. 2025/[40064737] Shanghai‐Kuwait 7000 km	Robotic‐assisted radical prostatectomy	Toumai robotic surgical system (TRSS)	1	0	None reported
Zhang et al. 2025/[40176186] Gansu provincial hospital‐Yangzhou University > 1700 km Gansu provincial hospital to branch Gansu hospital 70 km apart	5G remote surgery robot‐assisted gastrectomy	Toumai robotic surgery system (MicroPort medical robotics Co. Ltd.)	Long‐distance: 1 Short distance: 1	0	No postoperative complications or any adverse events
Liang et al. 2025/[40258794] Hangzhou, Zhejiang‐alaer, Xinjiang 4600 km	5G‐ultra‐long‐distance robotic hepatectomy	5G ultra‐remote four‐arm endoscopic robot surgery system	5	0	Postoperative complications, including 1 case of hypoproteinemia, and 1 case of pleural effusion. No severe complications
Wu et al. 2025/[40281540] Shanghai to shenzhen	5G remote robotic thyroidectomy	Tumai MT‐1000	1	0	No complications
Aldousari et al./[40736886] Shanghai to Kuwait 7000 km	Fiberoptic and 5G 3 prostatectomies, 2 partial nephrectomies	MicroPort Toumai	5	0	No complications
Wang et al. 2026/[41605542] Beijing to Hangzhou (1300 km), Harbin (1250 km), Urumqi (2800 km), Hefei (1000 km)	Randomized trial of local versus telesurgery for prostatectomy and partial nephrectomy	Multiple robotic platforms	72	0	No mortality, No readmission, No transfusion Non‐inferiority of telesurgery

Abbreviations: C: Conversion; N: Number of cases.

After review of available rRAS data, participants of the consensus conference endorsed the following statement: “Remote robotic surgery for many different specialties is technically feasible and can be as safe as non‐remote surgery when conducted by expert teams with appropriate infrastructure.”

Telementoring (telephone or web‐based surgical consultation, including group chats) has been used for open surgery [45, 46], and is officially approved in Canada, with an established reimbursement pathway [47]. Based on these data the panel endorsed the statement: “Remote teleproctoring, telementoring/teleprecepting without instrument control is feasible and could be a standard practice. Remote telementoring/teleprecepting with instrument control requires additional studies to assess and document routine reliability. It also requires delineation of qualifications and responsibilities.”

Remote robotic‐assisted co‐surgery includes operations in which a defined portion of the operation is performed from a remote location, either by a surgeon with unique expertise within a surgical discipline or by a surgeon from a different surgical discipline. The panel also discussed remote robotic co‐surgery with planned conversion, such as extracorporeal reconstruction following robotic cystectomy or colectomy. Finally, the key issues related to full rRAS, including robotic control, operating authority, responsibility over the patient, and emergency response, were discussed.

The expert consensus panel agreed that team training within the remote and the bedside teams is essential, as is joint training and rehearsal involving both teams together; and that clearly defined roles, responsibilities, and accountability frameworks are essential to ensure patient safety and equitable allocation of liability. The 2 recommendations made by Panel 1 are listed in Table 2.

### 3.2 PANEL 2: Clinical Pathways

Panel 2 also reviewed the literature describing the various rRAS operating models, and agreed that prior to the procedure, a consultation with the patient, including a full explanation of the planned workflow and responsibilities, occurs, as well as a process to ensure that all needed instruments are available and functional, is essential. The 2 recommendations made by Panel 2 are listed in Table 2.

### 3.3 PANEL 3: Emergency Management in Remote Robotic Surgery

All conference participants agreed that the greatest concern in removing the operating surgeon from the patient's operating room is patient safety.

The panel discussed varying levels of procedural risk. Factors that may inform the risk of remote procedures are outlined in Table 5. For many procedures, the complexity of the procedure is well defined, and validated scoring systems exist to define this complexity [48]. The panel unanimously agreed that comprehensive pre‐operative assessment of all these factors is essential (Table 6).

**TABLE 5:** Considerations for determining complexity of a remote robotic‐assisted operation.

Complexity of procedure Duration of procedure Number of surgical teams Risk of hemorrhage Risk of conversion Distance of remote surgery Major borders crossed by remote procedure Technical performance of network Technical performance of robot

**TABLE 6:** Key elements of a preoperative briefing.

Confirmation of working robotic and communication systems at both patient and remote surgeon sites Verify emergency communication protocol with remote and onsite teams Determine patient/procedure risk classification Confirmation of human and material resources as specified by risk classification Define key decision maker for each type of emergency Verify transfer protocol in non‐referral hospital settings Assess risk of major bleeding – verify concordance between remote surgeon and bedside clinician Verify site technical support– on‐site biomedical engineer and bedside clinician

The group then discussed conversion rates and transfusion rates for several commonly performed robotic procedures (Table 7). Certain procedures have low risks for both outcomes. For cholecystectomy, conversion rates in most large series are < 2% [49], and transfusion rates are also < 2% [50]. For prostatectomy, even lower rates of conversion (< 1%) [18, 51], and transfusion (< 1.5%) [18] have been reported. In contrast, pancreaticoduodenectomy shows higher risk with 10% conversion rates [52] and transfusion occurring in > 10% of patients [53]. All panel participants agreed that preparation for conversion or transfusion and staffing for patient‐site support should be tailored to procedure‐specific risk differences. To communicate the level of risk, the panel recommended that each procedure type be classified as high, medium, or low risk based on major bleeding risk, patient health status, and technology‐readiness assessment. High‐risk procedures are defined as those with *a* > 1.5% risk of major bleeding and requiring a blood transfusion [54]. Procedures with a risk between 0% and 1.5% are considered low‐to‐moderate risk, while those with approximately 0% risk are categorized as minimal risk.

**TABLE 7:** Conversion rates and transfusions rates of common robotic‐assisted operations.

Procedure	Studies/[PMID]	N robotic	Conversion rate (%)	Transfusion rate (%)	Note
Cholecystectomy	Tang et al. 2025 [40419930]	75,866	—	—	Meta‐analysis 17 studies
					RAC safe
	Lunardi et al. 2024 [38446451]	793,800	1.7	—	Retrospective cohort study
					Lower risk than LS
	Maegawa et al. 2025 [39277483]	59,216	0.8	—	US NSQIP database study
	Campbell et al. 2023 [38037087]	233,945	1.9	2.0	PINC AI healthcare Database study
Prostatectomy	Wang et al. 2023 [37721644]	6695	—	1.3%	Meta‐analysis 21 studies
	Li et al. 2025 [40586932]	130	0	0	SHURUI system, meta‐analysis 7 studies
	Lai et al. 2020 [32420205]	145	0	0	SP robot, meta‐analysis 12 studies
	Kulis et al. 2024 [38244127]	375	0.5	0	Senhance robot, 2 center study
	Hu et al. 2024 [39312046]	399	0	0	Hugo robot, meta‐analysis 6 studies
Colectomy	Solaini et al. 2022 [35650261]	13,506	6.8	—	Left colon, meta‐analysis 11 studies
	Alnumay et al. 2024 [39003434]	5238	5.8	—	Right colon, NSQIP study
	Tshann et al. 2022 [35566512]	1842	6.1	—	Right colon, meta‐analysis 25 studies
	Zheng et al. 2023 [37184773]	2772	3.2	—	Right colon, meta‐analysis 42 studies
LAR	Mohammed et al. 2024 [39624560]	1499	3.4	—	Meta‐analysis, comparison to laparoscopic, 7 studies
	Wang et al. 2025 [40140799]	1304	0	—	Single center study, comparison to laparoscopic
	Matsuyama et al. 2021 [34553225]	20,220	0.7	1.3	Matched meta‐analysis, Japan NCDB
Gastrectomy	Du et al. 2024 [39419891]	20,894	0.8	—	Meta‐analysis 86 studies
	Takahashi et al. 2023 [37433916]	1301	7.1	—	US NCDB study
	Holguin et al. 2022 [35802942]	1288	7.8	—	US NCDB study
Nephrectomy	Wang et al. 2024 [39443332]	4056	4.17	—	Meta‐analysis, 128 studies
	Razdan et al. 2022 [34962141]	142,040	2.98	—	US NCDB study
	Fink et al. 2025 [40600077]	4756	2.2	0.3	US NSQIP database study
Hysterectomy	Lenfant et al. 2023 [37856058]	110,306	2.2	2.1	Meta‐analysis 24 studies
	Cusimano et al. 2019 [31082383]	10,800	5.5	4.1	Meta‐analysis 51 studies
	Ind et al. 2017 [28762635]	3830	3.7	2.9	Meta‐analysis 36 papers
Hepatectomy	Wang et al. 2025 [40164991]	3938	5.0	10.3	Meta‐analysis 26 studies, major and minor
	Ziogas et al. 2021 [32989544]	225	3.6	18.4	Meta‐analysis 7 studies
	Mao et al. 2023 [37720925]	796	4.6	12.2	Meta‐analysis 12 studies
					Major hepatectomy
Whipple	Ouyang et al. 2022 [35280811]	1149	12	10.2%	Meta‐analysis 9 studies
	Kossenas et al. 2025 [40042699]	1964	10	13	Meta‐analysis 8 studies
	Tang et al. 2025 [39715160]	3695	8.5%	5.4	Meta‐analysis 10 studies

Abbreviations: LS: Laparoscopic surgery; NCDB: National Clinical Database; NSQUIP: National Surgical Quality Improvement Program; RAC: Robotic‐assisted cholecystectomy; SP: Single Port.

Medical and anesthetic assessment should include evaluation of the difficulty of airway control, the risk of hemodynamic instability unresponsive to standard resuscitation measures, and the risk of major intra‐operative cardiac events. High‐risk patients are defined as those classified as American Society of Anesthesiologists (ASA) Physical Status Class 4–5; moderate risk, ASA Class 3; and low risk, ASA Class 1–2. Technology‐readiness assessment should include the availability of backup power and communication networks. The 8 recommendations made by panel 3 are listed in Table 2.

### 3.4 PANEL 4: Technical Considerations in Remote Robotic Surgery

Panel 4 evaluated the technology requirements for safe and cybersecure rRAS. Recommendations were mainly based on technical guidelines published by an industry consortium (1), although input from surgeons and healthcare administrators added granularity to the details, while some were based on prior studies showing impact of latency on surgical precision and task completion during rRAS [2]. The 5 recommendations made by panel 4 are listed in Table 2.

### 3.5 PANEL 5: Medico‐Legal and Ethical Considerations in Remote Robotic Surgery

Because the patient may or may not have direct in‐person interactions with one or more of the responsible surgeons, panel 5 made 4 recommendations, which are listed in Table 2.

## 4 Discussion

Remote RAS has long been an aspiration of the surgical field. Advances in the architecture of many robotic systems combined with the increasing availability and affordability of high‐speed, secure telecommunications, now make this an achievable goal. At present, surgeons with advanced surgical and robotic expertise are already performing remote operations, primarily as demonstrations of feasibility, extending beyond preliminary studies.

Remote robotic surgery has clear potential to allow expert surgeons to operate from their home institutions to areas where experienced robotic surgeons may be scarce. Routine surgical procedures will likely increasingly be performed remotely for patients unwilling or unable to travel, helping to address the uneven geographic distribution of the surgical workforce and the growing shortage of surgeons. Importantly, rRAS is not limited to procedures within or across national borders but includes collaborations within a health care system in the same state, region, city, or even the same hospital. The purpose of these clinical guidelines is to help ensure that removing the operating surgeon from the patients' operating room is done with the least risk and the highest efficiency.

Our work builds up on previous such guidelines. A group of leaders from the Japanese Surgical Society developed surgical guidelines that included a useful glossary of terms, which has been expanded by subsequent contributors and further refined by our group (Supporting Information S1: Appendix Table A1). Consistent use of these terms is essential for clear communication regarding roles, performance, and workflows for clinical practice and future publications. Further, the Technical Considerations Panel (Panel 4) in our group expanded on landmark efforts of industry leaders in robotics and communications who previously assembled a framework of technical guidelines for rRAS (1) addressing network, communications, cybersecurity, and other technical considerations across clinical sites and during transmission. Several important principles emerged from and were strongly endorsed during the consensus conference. Foremost, all participants agreed that rRAS represents a unique workflow and must be studied and credentialed as such. Both teams at the remote physician site and the patient site (bedside) require appropriate staffing and specialized training. All agreed that training must extend beyond proficiency in performing RAS to include competencies specific to operating within a remote environment.

Communication was identified as central to the success of rRAS. Firstly, communication with patients should include a clear explanation of the rationale for performing remote surgery, available alternatives (e.g., patient travel to the primary surgeon's site), and any additional risks introduced by a remote workflow. Second, contingency plans for urgent and emergent situations during the remote workflow must be tailored to procedural risk, clearly communicated among all team members, and rehearsed through simulation. Third, communications on the day of surgery between the involved sites is particularly critical. This should include verification of adequate personnel, equipment functionality, and the speed and reliability of communications. A formal time‐out should verify the patient's identity, availability of imaging, equipment readiness, and contingency plans for conversion, transfusion, and emergencies.

Throughout the procedure, real‐time monitoring of network performance should be available. Prior to any high‐risk portions of the operation, such as resection or repair of major blood vessels, the surgeon should explicitly consider network performance when deciding whether to proceed.

Robotic surgery has grown rapidly over the past 3 decades and is now standard practice in many surgical procedures. There is no doubt that robotic technology has enabled many more patients and surgeons to benefit from the advantages of MIS. The field continues to evolve and is poised for further innovation in artificial intelligence (AI), automation, and remote workflows. While these three new frontiers in robotic surgery are advancing currently along largely independent paths, it is highly likely that progress in each field will accelerate advances in the others.

In the immediate future, efforts should focus on structured implementation of rRAS within regulated clinical environments, emphasizing standardized credentialing, team training, simulation‐based emergency preparedness, and prospective data captured through registries. In parallel, continued evaluation of network reliability, cybersecurity, and medico‐legal frameworks will be essential to support safe scale‐up. In the longer term, integration of advanced automation, AI–driven decision support, and semi‐autonomous functions is likely to further expand the indications for rRAS, improve procedural efficiency, and reduce geographic disparities in access to complex surgical care.

The clinical guidelines presented for rRAS represent an early effort by expert leaders and enthusiasts in this field to guide its safe and effective path forward. As with all consensus statements, this is a living document. Ongoing iterative updates of these recommendations will be necessary as technology, regulatory landscapes, and clinical evidence evolve, and future pioneer surgeons will undoubtedly build upon this foundation to further improve human health care.

## Author Contributions

**Yuman Fong:** conceptualization (lead), data curation (supporting), supervision (lead), project administration (equal), writing – original draft preparation (lead). **Dimitri A. Raptis:** conceptualization (supporting), data curation (lead), project administration (equal), formal analysis (lead), writing – review and editing (supporting). **Louis Kavoussi:** project administration (equal), formal analysis (supporting), writing – review and editing (supporting). **Sherry M. Wren:** project administration (equal), formal analysis (supporting), writing – review and editing (supporting). **Dennis Fowler:** project administration (equal), formal analysis (supporting), writing – review and editing (supporting). **Mehran Anvari:** project administration (equal), formal analysis (supporting), writing – review and editing (supporting). **Francesco Bianco:** project administration (equal), formal analysis (supporting), writing – review and editing (supporting). **Xiao Liang:** formal analysis (supporting), writing – review and editing (supporting). **Zheyong Li:** formal analysis (supporting), writing – review and editing (supporting). **Xiaoyu Yin:** formal analysis (supporting), writing – review and editing (supporting). **Micaela Piccoli:** formal analysis (supporting), writing – review and editing (supporting). **Saad Aldousari:** formal analysis (supporting), writing – review and editing (supporting). **John Marks:** formal analysis (supporting), writing – review and editing (supporting). **Baiyong Shen:** formal analysis (supporting), writing – review and editing (supporting). **Clifford Ko:** formal analysis (supporting), writing – review and editing (supporting). **Jelle Ruurda:** formal analysis (supporting), writing – review and editing (supporting). **Barbara Seeliger:** formal analysis (supporting), writing – review and editing (supporting). **Jeroen Hagendoorn:** formal analysis (supporting), writing – review and editing (supporting). **Rahila Essani:** formal analysis (supporting), writing – review and editing (supporting). **R. Matthew Walsh:** formal analysis (supporting), writing – review and editing (supporting). **Dmitry Oleynikov:** formal analysis (supporting), writing – review and editing (supporting). **Stephanie B. Jones:** formal analysis (supporting), writing – review and editing (supporting). **Vivek Bindal:** formal analysis (supporting), writing – review and editing (supporting). **Hyun Koo Kim:** formal analysis (supporting), writing – review and editing (supporting). **Woo Jin Hyung:** formal analysis (supporting), writing – review and editing (supporting). **Laleh Melstrom:** formal analysis (supporting), writing – review and editing (supporting). **Daniel Hashimoto:** formal analysis (supporting), writing – review and editing (supporting). **Francesco Celotto:** formal analysis (supporting), writing – review and editing (supporting). **Liaohai Leo Chen:** formal analysis (supporting), writing – review and editing (supporting). **Bryan M. Burt:** formal analysis (supporting), writing – review and editing (supporting). **Kelly Lafaro:** formal analysis (supporting), writing – review and editing (supporting). **Fernando Elli:** formal analysis (supporting), writing – review and editing (supporting). **Yusef Kudsi:** formal analysis (supporting), writing – review and editing (supporting). **Francesca Ratti:** formal analysis (supporting), writing – review and editing (supporting). **Erik Wilson:** formal analysis (supporting), writing – review and editing (supporting). **Paolo Pietro Bianchi:** formal analysis (supporting), writing – review and editing (supporting). **Annette Morgan:** formal analysis (supporting), writing – review and editing (supporting). **Stephen T. Bartlett:** formal analysis (supporting), writing – review and editing (supporting). **Laila Rashidi:** formal analysis (supporting), writing – review and editing (supporting). **Liu Yu:** formal analysis (supporting), writing – review and editing (supporting). **Bill Gawkins:** formal analysis (supporting), writing – review and editing (supporting). **Piet Hinoul:** formal analysis (supporting), writing – review and editing (supporting). **Bill Peine:** formal analysis (supporting), writing – review and editing (supporting). **Yanghee Woo:** formal analysis (supporting), writing – review and editing (supporting). **Mustafa Raoof:** formal analysis (supporting), writing – review and editing (supporting). **Daniel Jones:** formal analysis (supporting), writing – review and editing (supporting). **Supriya Deshpande:** project administration (supporting), formal analysis (supporting), writing – review and editing (supporting). **Jordana Bernard:** project administration (supporting), formal analysis (supporting), writing – review and editing (supporting). **Yulun Wang:** conceptualization (supporting), formal analysis (supporting), writing – review and editing (supporting). **Dieter C. Broering:** formal analysis (supporting), writing – review and editing (supporting). **Pier Cristoforo Giulianotti:** conceptualization (supporting), formal analysis (supporting), writing – review and editing (supporting).

## Funding

The authors have nothing to report.

## Conflicts of Interest

Yuman Fong: United States‐Israel Binational Science Foundation (Jerusalem, IL). Grant Number: 2007312; Research agreement with Imugene; Receive royalties from Imugene & Merck; Receives consulting fees from Medtronics, Covidien, Xdemics, Vergent Biosciences, Eureka Biologics, & Imugene; Serves on the advisory board of Sovato, Imugene; Family member is President and CEO of Xdemics. Louis Kavoussi: Serves on the advisory board of Sovato. Sherry M. Wren: Grants from Intuitive Surgical Foundation, Tsumura, USA Inc. *& World Journal of Surgery* (Editor in Chief: *World Journal of Surgery*); Receives consulting fees from Xpan Inc. & Intuitive Surgical; Received payment from the Writers Workshop at the ISW 2024 meeting; Receives payment from the Medical Board of California; Payment for attending meetings/travel from the International Society of Surgery (ISS) Société Internationale de Chirurgie (SIC); Leadership role on the American College of Surgeons board. Dennis Fowler: Financial support & stock options from Sovato. Mehran Anvari: Serves on the clinical advisory board of Insight Medbotics. Francesco Bianco: Serves on the advisory board of Sovato & receives stock options from Sovato. John Marks: Payment from ASCRS Annual Scientific Meeting, San Diego, California, May 10–13, 2025; Society of Robotic Surgery Annual Conference, Palais des Congrès, Strasbourg, France, July 16, 2025; Chilean Consensus on Early and Locally Advanced Rectal Cancer, August 21–23, 2025; ESCP 20th Scientific and Annual Conference. Palais des Congrès de Paris. September 10–12, 2025; Suntec Singapore, November 6–8, 2025; CRSA 16th Worldwide Congress. The Present and Future of Robotic Surgery Huntington beach California November 20–22, 2025; Scolla Summit Cirurgia Colorectal–Brazil–Virtual December 8, 2025. Financial support for attending meetings/travel from Main line Health. Serves as the James Widener Ray Chair of Colorectal Surgery; President of the International Society of Laparoscopic and Colorectal Surgeons; Member of Lankenau Institute for Medical Research scientists. Jelle Ruurda: Receives grants and fees for proctoring from Medtronic, Olympus, and Intuitive Surgical. Barbara Seeliger: Recipient of a grant from the French National Agency for Research (Agence Nationale de la Recherche, ANR) within the framework of the project AI‐DIAL (ANR‐22‐CE17‐0019‐01, ANR‐23‐IACL‐0004); Consultant agreement with Intuitive Surgical for the EAES robotics courses. Jeroen Hagendoorn: Receives payment as proctor for Intuitive Surgical. Rahila Essani: Receives consultant fees from Intuitive Surgicals, Conmed, & Virtamed. Dmitry Oleynikov: Chief Surgeon and Co‐Founder of Virtual Incision. Woo Jin Hyung: Research grants from Medtronic and GC Biopharma Corporation; Receives consulting fees from Ethicon & AstraZeneca; Serves of CEO of Hutom & receives stock options from Sovato. Daniel Hashimoto: Has grants/contracts from the American Surgical Association Foundation, the American Society for Gastrointestinal Endoscopy Intuitive Foundation, & Wellcome LEAP. Bryan M. Burt: Receives consulting fees from Medtronic, Intuitive Surgical, & AstraZeneca. Yusef Kudsi: Intuitive Hernia Research Grant; Receives consulting fees & payments from Gore and Intuitive; Holds patents issued related to digital sensors; Serves as the Secretary for the Clinical Robotic Surgery Association (CRSA). Erik Wilson: Receives consulting fees from CMR Surgical; Receives payments from Medtronic–Teaching/Research Intuitive–Teaching, Johnson & Johnson–Teaching/Research, Boston Scientific–Teaching/Research, USGI–Research; Serves as the Chief Medical Officer for GI Windows. Stephen T. Bartlett: Holds a grant from the Office of Naval Research with Medtronic, Sovato, & Virtual Incision as subcontractors on the grant. Laila Rashidi: Receives payment from Intuitive Surgical for teaching & training. Liu Yu: Employed by Microport Medbot. Bill Gawkins: Employed by Johnson & Johnson. Piet Hinoul: Employed by & has stock options from Virtual Incision. Bill Peine: Employed by Medtronic at the VP level & supported by the GRANT14143260, SC 1000020614, “Advanced Tele‐Robotic Surgery for Combat Casualty Care” from the Office of Naval Research. Receives payments for travel and expenses for role of VP of Research and Technology in Medtronic, & holds over 40 issued patents through Medtronic, with 50+ pending applications; & holds stock options in Medtronic. Yanghee Woo: Supported by the SU2C Grant Support of the Gastroesophageal Cancer Program Development Award #SU2C‐AACR‐30‐20‐237459. Receives consulting fees from Johnson & Johnson Ethicon; Serves on the advisory board of Imugene. Daniel Jones: Holds stock options from Health stock: Allurion stock. Jordana Bernard: Employed by & holds stock options from Sovato. Yulun Wang: Employed by Sovato & receives payments from Sovato for attending meetings/travel, serves on the board for Sovato, holds stock options from Sovato, & has other financial or non‐financial interests from Sovato. Pier Cristoforo Giulianotti: Supported by grants from Institutional training agreement with Intuitive for University of Illinois, Chicago. Receives consulting fees from Medtronic. Dimitri A. Raptis; Xiao Liang; Zheyong Li; Xiaoyu Yin; Micaela Piccoli; Saad Aldousari; Baiyong Shen; Clifford Ko; R. Matthew Walsh; Stephanie B. Jones; Vivek Bindal; Hyun Koo Kim; Laleh Melstrom; Daniel Hashimoto; Francesco Celotto; Liaohai Leo Chen; Kelly Lafaro; Fernando Elli; Francesca Ratti; Paolo Pietro Bianchi; Annette Morgan; Liu Yu; Bill Gawkins; Mustafa Raoof; Supriya Deshpande; Dieter C. Broering: These authors declare no conflicts of interest.

## Supporting information


Supporting Information S1

## Data Availability

All data has been presented in the manuscript and supplemental material.
