# Supplementary material for: Clinical Recommendations for Remote Robotic Assisted Surgery From the CRSA 2025 International Consensus Conference
Source: World J Surg. 2026 Jul 14;50(8):2274–88. doi: 10.1002/wjs.70468 (PMC13460931; doi:10.1002/wjs.70468)
Supplement: Supplementary file 1 [file WJS-50-2274-s001.docx]

**APPENDIX Table A1**

**Terms and Definitions***

*Bedside Proceduralist:* A practitioner credentialed to assist at the patient’s bedside during RAS performed by the primary surgeon at the RAS device console. Examples of practitioners who could be qualified to be a bedside proceduralist include a surgeon, physician, PA, NP, or RNFA with additional training to specifically assist during RAS procedures.

*Credentialed Practitioner:* A provider credentialed in accordance with the applicable healthcare system’s policies and procedures. Examples include a surgeon, physician, PA, or NP.

*Emergency Response Practitioner:* A credentialed practitioner who is available and qualified to address a surgical emergency at the patient site.

*Full Remote Robotic-Assisted Surgery:* A Remote Surgeon performs remote RAS as the primary surgeon who has full responsibility for the patient.

*Hard Stop:* A forced stoppage of workflow to determine the best next steps.

*Latency:* The time measurement between when a source transmits data and the destination receives the data.

*Patient Site:* The operating room or facility where the patient undergoes the remote RAS procedure.

*Patient Site Care Team*: The care team at the patient site supporting the remote procedure (e.g., nurses, OR technicians, anesthesiologists, clinical engineers, and other physicians).

*Primary Surgeon:* The credentialed practitioner who holds ultimate responsibility for the patient throughout the entire surgical process, including pre-operative, intra-operative, and post-operative care.

*Remote-enabled Robotic System*: This refers to the entire system used for remote procedures, including the robotic system at the patient site, the physician's console at the remote surgeon site, and any hardware or software needed to enable a safe and effective procedure over a surgical-grade network connection. Also known as “Remote RAS Device.”

*Remote-enabled Robot*: A RAS device that has been modified to enable its console to communicate with the bedside tower across a network.

*Remote Robotic-Assisted Co-Surgery*: A fully trained and credentialed Remote Surgeon collaborates with a fully trained Patient Site Credentialed Practitioner to complete a remote RAS procedure. Each surgeon/practitioner completes the part of the procedure for which they are credentialed.

*Remote Robotic-Assisted Surgery or Procedure:* A clinical procedure in which a physician uses a remote-enabled RAS device and surgical-grade network to perform a procedure on a patient in a different location. Also known as “remote RAS,” “remote surgery,” “remote procedure,” and/or “telesurgery.”

*Remote Surgeon*: A surgeon who is in a different location from the patient and Patient Site care team.

*Remote Surgeon Site*: The room or facility in which the remote surgeon performs remote RAS.

*Session:* This refers to a single instance of a remote RAS procedure encompassing the time from when the connection is established to when it is terminated.

*Surgical-Grade Network*: A managed network with high reliability, robust security, and guaranteed quality of service, which supports the requirements of remote procedures as outlined in the Expert Consensus-Based Technical Guidelines for Remote Surgery and Procedures.

*Telementoring/Teleprecepting without Instrument Control:* A fully trained and credentialed, as required, Remote Surgeon observes and mentors a Patient Site Credentialed Practitioner by providing verbal/visual guidance, including telestration. The Telementor has no control of the robotic instruments.

*Telementoring/Teleprecepting with Instrument Control:* A fully trained and credentialed Remote Surgeon provides a Patient Site Credentialed Practitioner with (1) supervisory and educational verbal-visual interactions, including telestration, and/or (2) guidance by taking control of the tools to demonstrate and/or complete a segment of the RAS procedure. Also referred to as Tele-Precepting in training scenarios.

*Teleoperation Latency:* The time between when the remote surgeon moves the input device to when that surgeon sees motion of the instrument on their monitor showing the video from the Patient Site robotic system.

*Telepresence*: The use of audio-video and tele-robotics technologies to enable a remote surgeon to be virtually in the operating room during remote RAS.

*Teleproctoring*: A qualified Remote Surgeon observes, evaluates and reports on the skills and performance of a Patient Site Practitioner.

*Time Out*: The surgical team's short pause, just before induction of anesthesia and/or just before incision, to confirm that all required equipment are available and functional, and that they are about to perform the correct procedure on the correct body part of the correct patient in the right facilities.

*Remote Surgery/Procedure Use Case:* The types or categories of procedures that practitioners perform as part of a remote surgery or remote procedure program, such as remote minimally invasive surgery, remote endovascular procedures, remote interventional procedures, remote endoscopy, or other diagnostic procedures that may be performed remotely. Telementoring with instrument control may be a component of any of these use cases.

*Adopted from Wang et al. [18] (PMID: 40581740)

**APPENDIX Table A2: Literature reviewed**

| **ARTICLE TYPE** | **AUTHOR/YEAR** | **PMID** |
| --- | --- | --- |
| **RECOMMENDATIONS** | | |
| **Delphi consensus** | Collins et al./2020 | 34337475 |
|  | Patel et al./2025 | 40167848 |
|  | Patel et al./2025 | 40643832 |
|  | Reddington et al./2026 | 40633604 |
| **Guidelines** | Mori et al./2024 | 38829562 |
|  | Wang et al./2025 | 40581740 |
|  | Carlos et al./2018 | 30376165 |
|  | Watkins et al./2025 | https://doi.org/10.1053/j.optechstcvs.2024.10.005 |
| **White paper** | Bogen et al./2019 | 30617422 |
|  | Ali et al./2024 | 38180541 |
| **Position paper** | De’Angelis et al./2022 | 35057836 |
|  | Somani et al./2025 | 40685283 |
| **Editorial/Commentary** | Alder et al./2020 | 32252161 |
|  | Cho et al./2020 | 32252162 |
|  | Larcher et al./2023 | 36243542 |
|  | Brownlee et al./2022 | 36509567 |
|  | Campbell et al./2023 | 37584680 |
|  | Sheetz et al./2024 | 38446439 |
| **Letter to Editor** | Ammendola et al./2023 | 37344319 |
|  | Daungsupawong et al./2024 | 39043508 |
|  | Feng et al. /2025 | 39643048 |
|  | Mao et al. /2024 | 38972827 |
|  | Moschovas et al./2024 | 38598833 |
|  | Zhou et al./2025 | 39793664 |
| **Expert opinion/News article/Perspective** | Moschovas et al./2025 | 39556854 |
|  | Patel et al./2024 | 38762391 |
|  | Chan et al./2024 | [ROBOTICS Robot to Open Conversion: Preparing the Team for the Unexpected - American Urological Association](https://auanews.net/issues/articles/2024/february-extra-2024/robotics-robot-to-open-conversion-preparing-the-team-for-the-unexpected) |
|  | Ackah et al./2021 | 33098967 |
| **Correspondence** | Patel et al./2024 | 38819496 |
|  | Saikali et al. /2024 | 39499368 |
|  | Saikali et al./2025 | 40580207 |
|  | Shafeea et al./2025 | 40768173 |
|  | Moschovas et al./2025 | 40128381 |
|  | Li et al./2023 | 35817641 |
| **MODELS/STIMULATION STUDIES** | | |
| **Trials** | Carneiro et al./2022 | 36173407 |
|  | Ebihara et al. /2023 | 36602754 |
|  | Jiang et al./2023 | 37845534 |
| **Feasibility study** | Morohashi et al./2023 | 37402741 |
|  | Wirz et al./2015 | 25599203 |
|  | Moustris et al./2023 | 37095315 |
|  | Yang et al./2024 | 38592415 |
| **Training/Protocol** | Xu et al./2014 | 24671353 |
|  | Kalipershad et al./2022 | 33947630 |
|  | Kim et al. /2024 | 38934235 |
|  | Almeida et al./2022 | 35998074 |
|  | Bludevich et al./2024 | 39144341 |
|  | Melnyk et al./2022 | 34387245 |
|  | Nakauchi et al./2022 | 36239792 |
|  | O’Sullivan et al./2016 | 27126584 |
| **Scientific abstract** | Kenawy et al./2020 | https://doi.org/10.1016/j.jamcollsurg.2020.08.338 |
| **PRECLINICAL STUDIES** | | |
| **Canine** | Aghanouri et al./2024 | 38536715 |
|  | Xu et al. /2024 | 38742557 |
| **Porcine** | Chia et al./2024 | 39806737 |
|  | Fan et al./2023 | 36691584 |
|  | Marescaux et al./2001 | 11574874 |
|  | Morohashi et al./2023 | 37402741 |
|  | Moschovas et al./2025 | 40105708 |
|  | Moschovas et al./2025 | 40112129 |
|  | Nakauchi et al./2022 | 36239792 |
|  | Nguan et al./2008 | 18803341 |
|  | Nguan et al./2008 | 18265415 |
|  | Oki et al./ 2023 | 37126192 |
|  | Ota et al./2024 | 37653350 |
|  | Patel et al./2024 | 39502103 |
|  | Rayman et al./2007 | 17554810 |
|  | Sterbis et al./2008 | 18295861 |
|  | Su et al./2018 | https://doi.org/10.1177/1687814018761921 |
|  | Takahashi et al. /2024 | 38071250 |
|  | Yang et al. /2024 | 38592415 |
|  | Zheng et al. /2020 | 32700149 |
| **HUMAN STUDIES** | | |
| **Clinical trial** | Wang et al./2026 | 41605542 |
|  | Dong et al./2024 | 39333992 |
|  | Liang et al./2026 | 40381824 |
|  | Feng et al./2022 | 36087608 |
| **Outcomes study** | Aldousari et al./2025 | 40064737 |
|  | Anvari et al./2005 | 15729068 |
|  | Boga et al./2021 | 33058376 |
|  | Bove et al./2003 | 12803985 |
|  | Ebihara et al./2024 | 37727067 |
|  | Fan et al./2023 | 36691584 |
|  | Gargollo et al./2019 | 29906433 |
|  | Marescaux et al./2001 | 11574874 |
|  | Moschovas et al./2024 | 39226445 |
|  | Patel et al./2019 | 31709402 |
|  | Tian et al./2020 | 32252160 |
|  | Wang et al./2024 | 37977963 |
|  | Yang et al./2024 | 38238690 |
|  | Yang et al./2022 | 35476791 |
|  | Zhang et al./2022 | 34996124 |
|  | Zhang et al./2025 | 40176186 |
|  | Zhou et al./2025 | 39557365 |
| **Retrospective cohort study** | Shah et al. /2022 | 35141775 |
|  | Campbell et al./2023 | 38037087 |
|  | Cao et al./2019 | 31255610 |
|  | Ceccarelli et al./2024 | 39154016 |
|  | Chen et al./2021 | 33111192 |
|  | Chiow et al./2021 | 34750608 |
|  | Chong et al./2022 | 36542135 |
|  | Connors et al./2025 | 40202592 |
|  | Curfman et al./2023 | 37225935 |
|  | Davis et al./2023 | 35927351 |
|  | Ferrari et al./2025 | 40698773 |
|  | Fink et al./2025 | 40600077 |
|  | Geitenbeek et al./2025 | 40282444 |
|  | Graaf et al./2024 | 39342074 |
|  | Herrera et al./2022 | 36509569 |
|  | Hoen et al./2021 | 32314230 |
|  | Holguin et al./2022 | 35802942 |
|  | Huy et al./2025 | 40905966 |
|  | Isiktas et al./2024 | 37842936 |
|  | Jones et al./2014 | 24937481 |
|  | Kawka et al./2025 | 40113617 |
|  | Kenigsberg et al./2021 | 33081512 |
|  | Klein et al./2024 | 39737144 |
|  | Kudsi et al./2021 | 33135558 |
|  | Kulis et al./2024 | 38244127 |
|  | Larach et al./2023 | 37284889 |
|  | Latif et al./2024 | 38896861 |
|  | Laquinandi et al./2024 | 38809354 |
|  | Leplo et al./2024 | 39013674 |
|  | Liu et al./2023 | 37058429 |
|  | Lunardi et al./2024 | 38446451 |
|  | Maegawa et al./2025 | 39277483 |
|  | Maertens et al./2022 | 35342848 |
|  | Matsuyama et al./2021 | 34553225 |
|  | Masetti et al./2022 | 35099776 |
|  | Palaiologos et al./2025 | 40842222 |
|  | Parenete et al./2024 | 39278764 |
|  | Ramesmayer et al./2023 | 36882812 |
|  | Razdan et al./2022 | 34962141 |
|  | Rhodes et al./2025 | 40182833 |
|  | Robinson et al./2022 | 33760973 |
|  | Sijberden et al./2024 | 38482665 |
|  | Sakakura et al./2022 | 34849975 |
|  | Saleh et al./2024 | 38879668 |
|  | Samreen et al./2019 | 29696591 |
|  | Sebastian et al./2026 | 40653401 |
|  | Servais et al./2022 | 34921815 |
|  | Sharma et al./2023 | 37193626 |
|  | Stefanishina et al./2025 | 40151771 |
|  | Stefanova et al./2024 | 38955837 |
|  | Takahashi et al./2023 | 37433916 |
|  | Tao et al./2021 | 33400103 |
|  | Toti et al./2025 | 40893753 |
|  | van Bodegraven et al./2024 | 38498397 |
|  | Alnumay Abdulaziz/2024 | 39003434 |
|  | Al-Temimi et al./2019 | 31230107 |
|  | Aziz et al./2022 | 33409592 |
|  | Emile et al./2023 | 37075480 |
|  | Wang et al./2025 | 40140799 |
|  | Zhang et al./2020 | 32676411 |
| **Prospective study** | Anderson et al./2020 | 31076952 |
|  | Brunaud et al./2008 | 19041009 |
|  | Hudolin et al./2023 | 37452580 |
|  | Milone et al./2024 | 38835071 |
|  | Staib et al./2025 | 40632309 |
|  | Stephan et al./2021 | 33513657 |
|  | Zattoni et al./2017 | 28827196 |
| **Case report/Brief report** | Bauer et al./2001 | 11886670 |
|  | Conticchio et al./2023 | 37222177 |
|  | Fan et al./2025 | 39744935 |
|  | Felli et al./2014 | 24791165 |
|  | Rogers et al./2024 | 39460826 |
|  | Sudan et al./2012 | 27638285 |
|  | Tian et al./2024 | 39831214 |
|  | Wu et al./2025 | 40281540 |
| **ANALYSIS** | | |
| **Systematic review & Metanalysis** | Balduzzi et al./2021 | 33301071 |
|  | Cusimano et al./2019 | 31082383 |
|  | Du et al./2024 | 39419891 |
|  | Liu et al./2023 | 37058429 |
|  | Lingbo et al./2025 | 41024011 |
|  | Luciani et al./2022 | 33743146 |
|  | Junjei Hu et al./2020 | 32400946 |
|  | Ind et al./2017 | 28762635 |
|  | Genova et al./2021 | 32902707 |
|  | Hinojosa-Gonzalez et al./2022 | 35616429 |
|  | Kadam et al./2022 | 35393759 |
|  | Kossenas et al./2025 | 40042699 |
|  | Kossenas et al./2025 | 39899136 |
|  | Lenfant et al./2023 | 37856058 |
|  | Hengrui Liang et al./2018 | 28628562 |
|  | Ma et al./2021 | 33941112 |
|  | Martino et al./2022 | 35644881 |
|  | Mohammed et al./2024 | 39624560 |
|  | Panin et al./2023 | 37779579 |
|  | Ramadan et al./2025 | 40619483 |
|  | Rudiman et al./2023 | 37663974 |
|  | Squillaro et al./2023 | 37561350 |
|  | Solaini et al./2022 | 35650261 |
|  | Tang et al./2021 | 33536032 |
|  | Tschann et al./2022 | 35566512 |
|  | Chong‑jian Wang et al./2024 | 39443332 |
|  | Wang et al./2025 | 40140799 |
|  | Zhao et al./2024 | 39042305 |
|  | Zhang et al./2022 | 35494020 |
|  | Zheng et al./2023 | 37184773 |
| **Systematic review** | Barba et al./2022 | 35246740 |
|  | Bianchi et al./2022 | 36061042 |
|  | Coco et al./2025 | 40908371 |
|  | Frenkel Catherine/2023 | 37040578 |
|  | Ibrahim et al./2025 | 40884599 |
|  | Kourounis et al./2024 | 38721875 |
|  | Lechien et al./2023 | 37038204 |
|  | Muaddi et al./2021 | 32398482 |
|  | Reddy et al./2025 | 39435556 |
|  | Veneziano et al./2020 | 31701210 |
|  | Morais et al./2025 | 40767931 |
|  | Reinisch et al./2023 | 35727485 |
|  | Chu et al. /2025 | 40229598 |
| **Review** | Berber et al./2016 | 26858014 |
|  | Ceccarelli et al./2020 | 32487136 |
|  | Chan et al./2024 | 38914507 |
|  | Dohler et al./2024 | 39549181 |
|  | Fan et al./2025 | 39744935 |
|  | Felli et al./2014 | 24791165 |
|  | Gamal et al./2024 | 38231279 |
|  | Godley et al./2025 | 39994045 |
|  | Kim et al./2024 | 39828385 |
|  | Li et al./2024 | 39527379 |
|  | Moschovas et al./2025 | 40037973 |
|  | Pandav et al./2022 | 35266317 |
|  | Patel et al./2024 | 38231309 |
|  | Pfennig et al./2022 | 35545476 |
|  | Rocco et al./2024 | 38833111 |
|  | Sheetz et al./2020 | 31922557 |
|  | Shehata et al./2025 | 40619175 |
|  | Soyama et al./2024 | 38801309 |
|  | Stahel et al./2022 | 35135584 |
|  | Xia et al./2021 | 33744069 |
|  | Xie et al./2025 | 40535085 |

**APPENDIX Figure A1**. The Danish Consensus Process Adopted and modified from Pollock et. al. (PMID: 36495912)
